# Antimicrobial mechanism of theaflavins: They target 1-deoxy-D-xylulose 5-phosphate reductoisomerase, the key enzyme of the MEP terpenoid biosynthetic pathway

**DOI:** 10.1038/srep38945

**Published:** 2016-12-12

**Authors:** Xian Hui, Qiao Yue, Dan-Dan Zhang, Heng Li, Shao-Qing Yang, Wen-Yun Gao

**Affiliations:** 1 Key Laboratory of Resource Biology and Biotechnology in Western China (Ministry of Education), College of Life Sciences, Northwest University, 229 North Taibai Road, Xi’an, Shaanxi 710069, China

## Abstract

1-Deoxy-D-xylulose 5-phosphate reductoisomerase (DXR) is the first committed enzyme in the 2-methyl-D-erythritol 4-phosphate (MEP) terpenoid biosynthetic pathway and is also a validated antimicrobial target. Theaflavins, which are polyphenolic compounds isolated from fermented tea, possess a wide range of pharmacological activities, especially an antibacterial effect, but little has been reported on their modes of antimicrobial action. To uncover the antibacterial mechanism of theaflavins and to seek new DXR inhibitors from natural sources, the DXR inhibitory activity of theaflavins were investigated in this study. The results show that all four theaflavin compounds could specifically suppress the activity of DXR, with theaflavin displaying the lowest effect against DXR (IC_50_ 162.1 μM) and theaflavin-3,3′-digallate exhibiting the highest (IC_50_ 14.9 μM). Moreover, determination of inhibition kinetics of the theaflavins demonstrates that they are non-competitive inhibitors of DXR against 1-deoxy-D-xylulose 5-phosphate (DXP) and un-competitive inhibitors with respect to NADPH. The possible interactions between DXR and the theaflavins were simulated via docking experiments.

Up to date, 2-methyl-D-erythritol 4-phosphate (MEP) pathway for the biosynthesis of terpenoids has been found and established^1^. Research has shown that this terpenoid biosynthetic route is essential for the survival of most bacteria, including human pathogens, but is absent in mammals and humans^1^. The alternative pathway has thus been considered an attractive target for the screening of novel antibacterial agents. 1-Deoxy-D-xylulose 5-phosphate reductoisomerase (DXR), the first committed enzyme of the 2-methyl-D-erythritol 4-phosphate (MEP) pathway that catalyzes the rate-limiting conversion of 1-deoxy-D-xylulose 5-phosphate (DXP, **1**, Fig. 1) to MEP (**2**), has been accepted as one of the most promising targets in the search for antibiotics^1^,^2^. Much research has therefore been performed to seek its inhibitors, resulting in the discovery of fosmidomycin (**3**, Fig. 1), a phosphonate compound previously isolated from *Streptomyces lavendulae* and its structural analogue FR900098 (**4**). These two highly hydrophilic compounds are not only potent DXR inhibitors, but show strong antibacterial effects as well^3^. Clinical data show that **3** is somewhat effective in treating malaria caused by *Plasmodium falciparum*^4^. Kaiser *et al*. have found that the leaf extracts of *Cercis siliquastrum*, a Mediterranean plant, exhibit strong inhibitory activity against DXR^5^.

As the most popular functional drink worldwide, tea has aroused widespread interest due to its potential benefits to human health^6^,^7^. Theaflavins, polyphenolic compounds isolated from fermented tea, are the products of the postharvest enzyme-mediated fermentation of tea leaves^8^. Four theaflavin polyphenols, theaflavin (TF), theaflavin-3-gallate (TF3G), theaflavin-3′-gallate (TF3′G), and theaflavin-3,3′-digallate (TF3,3′G) (Fig. 2) have been characterized. Research has shown they exhibit a broad spectrum of physiological effects, such as anticlastogenic, antivirus, anticancer, and antibacterial activities^9^,^10^,^11^, with their antibacterial property being especially emphasized^12^,^13^. However, as yet, little has been reported on the modes of action of the theaflavins. We therefore tested the inhibitory effect of the theaflavins on the activity of *E. coli* DXR. The aims of the study are to disclose the possible antibacterial mechanism of the theaflavins and to seek new DXR inhibitors.

## Results

### Stability of the theaflavins

The theaflavins are unstable compounds, especially under a basic condition^14^. Because the DXR inhibition assay needs to be carried out in 50 mM Tris-HCl buffer at pH 7.4 and incubated at 37 °C for 30 min, we have to test whether the theaflavins can survive the assay condition, although it is almost neutral. The compounds were actually incubated at 37 °C for 35 min, 5 min longer than that of the real DXR assay. The results, as depicted in Table 1, indicate that almost half of the theaflavins decomposed after incubation. That is to say that these compounds are unstable even under the weak basic condition. To stabilize them, we added ascorbic acid (VC) (final concentration 2 mM) to the assay mixture because it is a highly effective antioxidant and often used as a protective agent. The results (Table 1) show that the decomposition of the theaflavins was almost completely suppressed in the presence of VC (The HPLC profiles see also Fig. S1 in the Supplementary Material). Thus VC (2 mM) was used to protect the theaflavins in the following assays.

### Evaluation of the pre-column derivatization HPLC method for its reliability in this study

**Figure 3:**
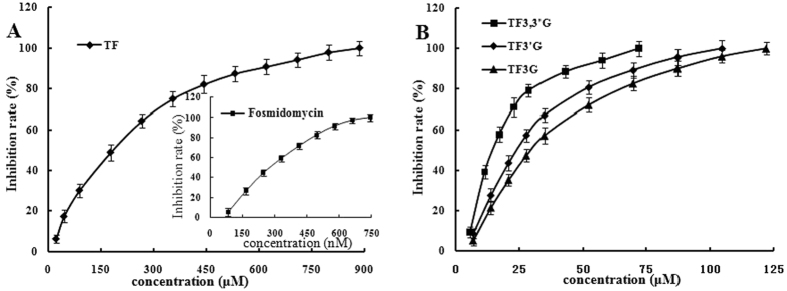
Inhibition curves of the theaflavins against DXR. (**A**) Inhibition curve of TF (inset: inhibition curve of fosmidomycin); (**B**) Inhibition curves of TF3G, TF3′G, and TF3,3′G.

We evaluated the reliability of the HPLC method in the measurement of DXR inhibitory activity of the theaflavins. The results show that use of VC and the theaflavins would not interfere with the quantification of DXP (for 2,4-dinitrophenylhydrazine (DNPH) derivatization and HPLC profiles, see Figs S2 and S3 in the Supplementary Material). Additionally, we determined the inhibition curve of fosmidomycin against DXR using the HPLC method, and the results show that it could inhibit DXR in a clear dose-dependent manner (Fig. 3A, the insert) with an IC_50_ of 0.27 μM. These data are in good agreement with the previously published results^5^,^15^.

### Determination of inhibition of the theaflavins against alkaline phosphatase (AP)

**Figure 4:**
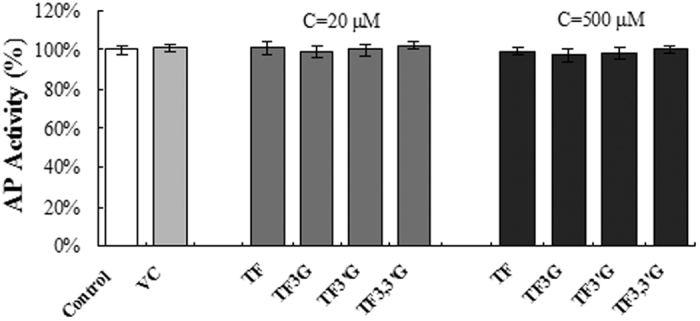
Determination of inhibition of VC and the theaflavins against AP. The reaction mixtures comprise 10 μL AP buffer and 2 U AP in a final volume of 100 μL, DXP was added (final conc. 1 mM) to start the reaction. The reaction mixtures were incubated at 37 °C for 120 min and subsequently derivatized with DNPH and analyzed with the HPLC method. For VC assay, 2 mM VC was added before the addition of DXP. For assays of the theaflavins, the compounds were added separately before the addition of DXP (two concentrations, 20 and 500 μM for each compound were applied).

The other factor that could influence the determination of inhibitory activity of the theaflavins against DXR would be the AP because we must use it to dephosphorylate DXP before DNPH derivatization. We thus measured whether VC and the theaflavins might display any inhibition against this enzyme. We find that VC has no effect on the activity of AP at 2 mM and the four theaflavins also have no inhibition against AP at all (Fig. 4), even at final concentrations as high as 500 μM.

### The inhibitory activity of the theaflavins against DXR

With the validated HPLC method, we also evaluated whether VC could affect DXR activity. The result shows that 2 mM VC had no effect on the activity of DXR. Then, we evaluated the DXR inhibitory activity of all four theaflavins at a final concentration of approximately 150 μM, employing 2% DMSO as a co-solvent and VC as a protective agent. As expected, the positive control fosmidomycin showed complete inhibition of DXR activity at 1.0 μM. The results reveal as well that all of the compounds were active in suppressing DXR activity at the selected concentration: TF exhibited approximately 40% inhibition against DXR, whereas the other three completely inhibited the activity of the enzyme. Encouraged by these experiments, we further determined the DXR inhibitory effects of the theaflavins at different concentrations. From the results depicted in Fig. 3, we are able to observe that all of the theaflavins displayed clear concentration-dependent inhibitory activities against DXR. Their IC_50_ values could thereby be calculated and listed in Table 2. We can find that amongst the four compounds, TF displays the lowest effect against DXR with an IC_50_ of 162.1 μM and TF3,3′G exhibits the highest (IC_50_ 14.9 μM).

It has been reported recently that some flavonoids could produce promiscuous inhibition against DXR when using DMSO as a co-solvent^16^. Triton X-100 is often used as a dispersing agent to discriminate between the specific and non-specific inhibitions^17^,^18^,^19^. We therefore determined the DXR inhibitory activity of the theaflavins using 0.01% Triton X-100 as a surfactant to verify whether the theaflavins were aggregating inhibitors of DXR. Exactly the same procedure as described in the experimental part was followed to carry out the measurements. We first tested the influence of Triton X-100 on DXR and found that the detergent had no effect on the activity of DXR at a concentration of 0.1%. Then, we set two distinct concentrations for each of the theaflavins to evaluate its inhibition against DXR, one was close to its IC_50_ value and the other was about twice of it. We also selected baicalein as a positive control since it has been demonstrated to be a non-specific inhibitor of DXR^16^. The data shown in Table 3 exhibit that for all four theaflavins, no noticeable difference in inhibition could be observed in the presence and absence of the detergent at either concentration, but for baicalein, the presence of Triton X-100 could significantly reduce its inhibition potency. Therefore, in light of the criteria established by Shoichet and co-workers^20^, these observations imply that the theaflavins could inhibit DXR via a specific mechanism.

### Particle size analysis by dynamic light scattering (DLS)

DLS is a widely utilized technique in materials science to measure particle sizes in solutions^20^,^21^. In this study, we used the method to detect aggregates in the samples in which each of the four theaflavins was diluted to an end concentration of about 3–5 times its IC_50_ value in tris-HCl buffer (50 mM, pH 7.4), in the absence or presence of DXR (4 μg/mL). Baicalein was referenced as a positive control owing to the same reason as mentioned above. The particle size evaluation was performed by a DLS analyzer and the results display that except for baicalein, the theaflavins produce no detectable particles. The data are shown in Table 4.

### Determination of the inhibition kinetics of the theaflavins against DXR

**Figure 5:**
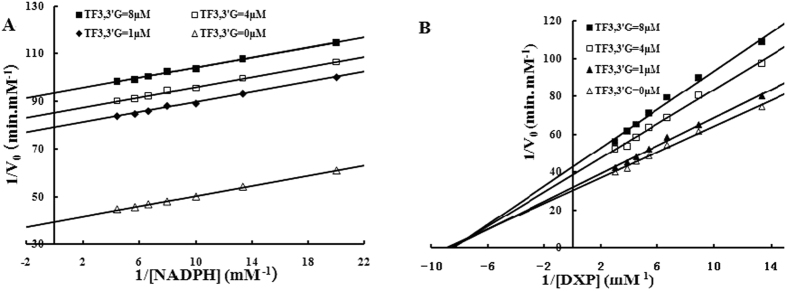
Lineweaver–Burk plots of DXR with respect to NADPH (**A**) or DXP (**B**) in the absence and presence of TF3,3′G. Assay mixtures comprised 50 mM Tris-HCl (pH 7.4), 5 mM MgCl_2_, 2 mM VC, TF3,3′G (0, 1, 4, or 8 μM), and 2 μg/mL DXR in a final volume of 100 μL. For **A**, the mixtures contained 1 mM DXP and NADPH (final conc. 0.05 to 0.275 mM); For **B**, the mixtures contained 0.5 mM NADPH and DXP (final conc. 0.075 to 0.3375 mM). The incubation was performed for 10 min at 37 °C before the reaction mixtures were hydrolyzed with AP, derivatized with DNPH, and analyzed using the HPLC method.

To determine the modes of the inhibitory action of the theaflavins against DXR, the initial enzyme kinetics were investigated over fixed inhibitor concentrations and at different DXP/NADPH concentrations employing the HPLC method described above. Lineweaver-Burk (LB) graphical charts were obtained by plotting the reciprocal of the reaction velocity against the reciprocal of the concentration of DXP/NADPH. The results, as shown in Fig. 5 (for TF3,3′G) and Figs S4 and S5 in the Supplementary Material, indicate that all four theaflavins are uncompetitive inhibitors of DXR when NADPH is the varied substrate and produce noncompetitive patterns against DXP. The *K*_i_ values against NADPH and DXP were calculated accordingly and are listed in Table 2.

### The results of docking experiment

The molecular docking results evidenced that both compounds locate at the gateway of the hydrophobic pocket of DXR, next to the binding position of NADPH and quite far away from the DXP location (as shown in Fig. S6). Therefore, it could be deduced that the binding of the theaflavin compounds just outside the active pocket might result in the alteration of the DXR conformation, thus retarding the enzyme activity. The close-up views of the docking results (as depicted in Fig. S7) show the interactions between the inhibitors and the substrates/residues. There are more wire balls and H-bonds in picture A than in B, indicating that compound TF3,3′G produces much stronger interactions with the target protein than compound TF does. Further analyses led to the below observations: i) in both pictures, no black wire ball is visible, indicating that neither of the two compounds has close contact with DXP. This result is consistent with those acquired in Fig. 5 and supports that the theaflavins are non-competitive inhibitors against DXP; ii) the binding sites for TF3,3′G include six residues: Lys36, Asp56, Val101, Gln81, Glu339, and Arg385. It forms three H-bonds with the Lys36, Asp56, and Glu339 residues (green bead wires) and produces close contact with the other threes (yellow wire balls). Two of the H-bonds are between the hydroxyl groups of the gallate side chains and the Asp56 and Glu339 residues, and the third is between its catechin hydroxyl group and Lys36. In addition, TF3,3′G forms another two H-bonds between its catechin hydroxyl groups and the pyrophosphate moiety of NADPH and has some close contact with NADPH (blue wire balls); iii) the compound TF binds at four residues: Val101, Gly102, Ala103, and Ala104. It forms two H-bonds with the Ala103 and Ala104 residues and generates close contact with the other two. Because TF has no gallate side chain, both of the H-bonds form through contact between its catechin hydroxyl groups and the residues. TF also forms two H-bonds with the pyrophosphate moiety of NADPH via its catechin hydroxyl groups on the other side and has some close contact with NADPH.

## Discussion

Fosmidomycin, hitherto the most potent inhibitor of DXR, has drawbacks such as poor bioavailability, short plasma half-life (~1 h), and metabolic liability, which have precluded its *in vivo* application as a DXR inhibitor^1^. There have been numerous reports on the antimicrobial effects of tea polyphenols^6^. With this in mind, we initiated a study to look for inhibitors of DXR protein in tea polyphenols, focusing on theaflavins, and also uncover the mode of their actions.

Having overcome the stability issue of the theaflavins under the DXR assay conditions and validated the HPLC method, we measured the inhibition of the tea polyphenols against DXR, and the data indicate that compound TF, lacking a gallate side chain, exhibits the lowest DXR inhibitory activity among the four theaflavins, with an IC_50_ larger than 100 μM, whereas the other three with at least one gallate side chain show stronger inhibition against the target than TF, with IC_50_ values in the range of 14.9 to 29.2 μM. Thus, the DXR-inhibitory activities of the theaflavins apparently correspond to the gallate side chain in the structure. The same phenomenon has been observed on the suppressive capacity of these compounds against *Bacillus cereus*^13^. Furthermore, all four compounds suppress the activity of DXR in clear dose-dependent manners (Fig. 3).

Although there have been many publications on the bioactivities of flavonoids^6^,^22^, the promiscuous inhibition of this type of compound against various proteins has been extensively reported^17^,^20^,^21^,^23^,^24^,^25^. The non-specific suppression of DXR by several flavonoids has also been observed^16^,^19^. The reason for this phenomenon is attributed to the interaction of flavonoids and enzymes to produce aggregates of enzymes, leading to decreased or even a loss of enzymatic activities^18^,^20^. To determine whether a compound is a promiscuous or aggregation-based inhibitor, Shoichet and co-workers put forward some criteria that have been accepted and adopted practically in inhibitor screening^20^.

From view of the chemical structures, the theaflavins are polyphenolic compounds containing a flavanol moiety. Accordingly, they could possibly exert promiscuous inhibition on DXR. We thus carried out extra assays to judge whether the inhibitory manner of the theaflavins is specific, under the guidance of Shoichet’s criteria^20^. First, we added Triton X-100 to the assay mixtures to determine if the inhibition produced by the theaflavins was sensitive to addition of the detergent. Second, we analyzed the reaction mixtures with DLS to verify whether there were detectable particles formed in the systems. Because both of these extra experiments gave negative results, we conclude that the tested compounds truly produced specific inhibition against DXR. Our data also show that the theaflavins have no inhibitory capacity against AP, a completely unrelated enzyme to DXR. This observation could support the specific inhibition manner of the theaflavins against DXR.

Having elucidated that the theaflavins exerted specific suppression against DXR, we determined their inhibition kinetics using Lineweaver-Burk double-reciprocal plots. The results show that all four theaflavins are non-competitive inhibitors of DXR with respect to DXP and un-competitive inhibitors with respect to NADPH. The non-competitive inhibition mode of the compounds against DXP is predictable because they share no structural similarity with DXP, and their un-competitive inhibition mode versus NADPH is reasonable because DXR has been determined to mediate the conversion of DXP to MEP via an ordered Ping Pong mechanism, with NADPH binding first^26^.

To obtain more information about the interaction between the theaflavins and DXR, we performed some docking experiments to simulate the binding modes of TF3,3′G and TF to the enzyme. The docking results support the conclusions we obtained from the other experiments. Furthermore, from the docking results we can conclude the interaction between TF3,3′G and DXR is much stronger than that between TF and the enzyme because there are more H-bonds and more close contact between TF3,3′G and DXR. This could not only explain why compound TF3,3′G is a much more potent inhibitor of DXR than TF but also accounts for the importance of the gallate side chain for the DXR inhibitory activity of the theaflavins.

Combining all the above observations, we could deduce that after NADPH binds to DXR, TF3,3′G (or another theaflavin compound) could specifically bind to the enzyme in the vicinity of the binding sites of NADPH, forming a complex that could retard the departure of the oxidized product NADP^+^, and thus suppressing the activity of the enzyme^26^.

In summary, the data obtained in this study show that the polyphenolic compounds theaflavins are a novel DXR inhibitory chemotype which can specifically inhibit the activity of DXR with weak to medium effect. Based on these data, we might only partially disclose the antibacterial mechanism of theaflavins. The modest DXR inhibition of these polyphenolic compounds may suggest that the theaflavins would exert their antibacterial effect also via suppressing the functions of other targets which need further elucidation.

## Materials and Methods

### Materials

Theaflavins were purchased from Chengdu Biopurify Phytochemicals, Ltd. (Chengdu, China), and stock solutions of them (10 mg/mL) were prepared in 40% aqueous DMSO or 0.1% Triton X-100 just before use. NADPH was purchased from GEN-VIEW SCIENTIFIC INC. (Tallahassee, FL, USA). Fosmidomycin was from Toronto Research Chemicals, Inc. (North York, ON, Canada). Calf intestine AP was purchased from Takara (Dalian, China). Triton X-100 and DNPH were purchased from Sinopharm Chemical Reagent Co., Ltd. (Beijing, China). DXP was synthesized according to a procedure previously reported^27^. The preparation of *E. coli* DXR was carried out in accordance with a published method^28^. HPLC grade methanol was purchased from Sigma-Aldrich Chemical Co. (Shanghai, China). All other chemicals are of analytical grade.

### Stability of the theaflavins under the DXR assay conditions

Stability of the theaflavins in Tris-HCl buffer was evaluated using an Agilent 1200 HPLC equipped with a DAD detector. The theaflavins were separately diluted into 50 mM Tris-HCl buffer (pH 7.4) containing 5 mM MgCl_2_ and 2% (W/V) DMSO to a final concentration of 100 μM in the absence and presence of 2 mM VC. The mixtures were subsequently incubated at 37 °C for 35 min before they were centrifuged at 6000 rpm for 3 min and analyzed. HPLC conditions: Column, Shim-pack VP-ODS column (250 × 4.6 mm, 4.6 μm). Detection wavelength: 280 nm. Injection volume: 20 μL. The mobile phase consists of 60% solvent A [2% acetic acid in water (v/v)] and 40% solvent B (acetonitrile). Flow rate: 0.7 mL/min. Column temperature: 25 °C.

### Determination of inhibitory activity of the theaflavins against *E. coli* DXR

The inhibitory activity of the theaflavins and baicalein against *E. coli* DXR was determined using the pre-column derivatization HPLC method published by this group with minor modifications^29^. The assay mixture consists of 50 mM Tris-HCl (pH 7.4), 5 mM MgCl_2_, 2 mM VC, 0.5 mM NADPH, 2% (W/V) DMSO or 0.01% Triton X-100, and 4 μg/mL DXR in a final volume of 100 μL. The theaflavin compounds or baicalein were added to the mixtures separately before the addition of DXP (final conc. 1 mM) to start the reaction. In a control assay, fosmidomycin (**3**) was used instead of the theaflavins. The reaction mixture was incubated at 37 °C for 30 min before the DXR was inactivated by boiling for 3 minutes. Subsequently, 10 μL AP buffer (1 M Tris-HCl, 1 M NaCl_2_ 50 mM MgCl_2_, pH 9.5) and 2 U AP were added, and the mixture was incubated at 37 °C for 120 min. 10 μL DNPH (120 mM in 30% perchloric acid, V/V) and 280 μL methanol were supplemented. After incubated at 37 °C for another 45 min, the mixture was centrifuged at 6000 rpm for 3 min and the supernatant was used for HPLC analysis on the Agilent 1200 HPLC system. HPLC conditions: Column, Shim-pack VP-ODS column (250 × 4.6 mm, 4.6 μm). Injection volume: 60 μL. Gradient: 0 min, 40% acetonitrile; 17 min, 80% acetonitrile; 18–20 min, 40% acetonitrile. Detection wavelength: 360 nm. Flow rate: 0.7 mL/min. Column temperature: 30 °C.

### Determination of AP inhibitory activity of the theaflavins

The HPLC method described above was also used to determine the inhibitory activity of the theaflavins against AP. The reaction mixture comprises 2 mM VC, 10 μL AP buffer and 2 U AP in a final volume of 100 μL. The theaflavin compounds (for each compound, two concentrations were applied, one around its IC_50_ value, and the other 500 μM) were added to the mixtures separately before the addition of DXP (final conc. 1 mM) to start the reaction. The reaction mixtures were incubated at 37 °C for 120 min, and the AP was inactivated in boiling water for 3 min. Then, the reaction mixture was derivatized with DNPH and analyzed with the HPLC method.

### Measurement of the particle size by DLS

The particle size of the theaflavin compounds and baicalein in Tris-HCl buffer (50 mM, pH 7.4) containing 2 mM VC and 2.0% DMSO (V/V) was measured in the absence and presence of DXR (final concentration: 4 μg/mL) on a DLS analyzer (NICOMP-380, Particle Sizing Systems, Inc., Santa Barbara, Calif., USA). The detector time was set to 10 min. The concentration of each of the theaflavins was 3–5 times of its IC_50_ value. A solution of 2.0% aqueous DMSO was used as a reference.

### Docking experiment

Autodock 4.2.6 software was used for docking experiments, and the results were shown by Chimera 1.10.1 software. The profiles of the crystal of the DXR-NADPH-Mg^2+^-fosmidomycin quaternary complex were obtained from Protein Data Bank (PDB accession code 2EGH). Fosmidomycin was removed, and subsequently DXP was docked into the binding sites of fosmidomycin because it had been suggested that DXP could be superposed exactly onto fosmidomycin^30^. Then, the docking simulation was carried out using the theaflavins as ligands and the mimic DXR-NADPH-Mg^2+^-DXP quaternary complex as a receptor.

## Additional Information

**How to cite this article**: Hui, X. *et al*. Antimicrobial mechanism of theaflavins: They target 1-deoxy-D-xylulose 5-phosphate reductoisomerase, the key enzyme of the MEP terpenoid biosynthetic pathway. *Sci. Rep.* **6**, 38945; doi: 10.1038/srep38945 (2016).

**Publisher's note:** Springer Nature remains neutral with regard to jurisdictional claims in published maps and institutional affiliations.

## Supplementary Material

Supplementary Information

## Figures and Tables

**Figure 1:**
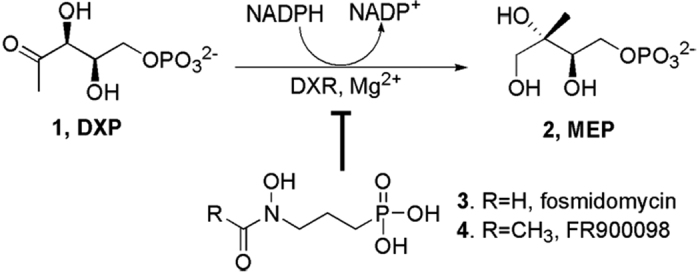
The first committed step of the MEP terpenoid biosynthetic pathway and its inhibitors.

**Figure 2:**
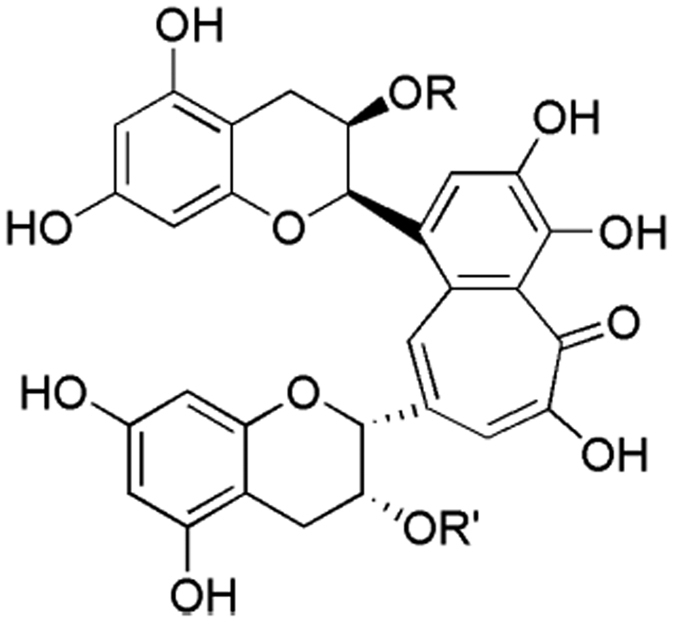
Structures of the theaflavins. R = R’ = H, theaflavin (TF); R = galloyl, R’ = H, theaflavin-3-gallate (TF3G); R = H, R’ = galloyl, theaflavin-3′-gallate (TF3′G); R = R’ = galloyl, theaflavin-3,3′-digallate (TF3,3′G).

**Table 1:** Stability of the theaflavins under assay conditions in the absence and presence of VC.

Entry	Concentration (μM)*			
	TF	TF3G	TF3′G	TF3,3′G
1	63.01 ± 3.73	60.88 ± 2.77	61.17 ± 4.29	55.26 ± 4.32
2	98.97 ± 3.27	98.15 ± 2.86	98.08 ± 3.02	96.72 ± 2.78

^*^The concentrations of the theaflavins before incubation are 100 μM each; Entry 1: The concentrations of the theaflavins after incubation in the absence of 2 mM VC; Entry 2: The concentrations of the theaflavins after incubation in the presence of 2 mM VC.

**Table 2:** IC_50_ values, inhibition kinetics, and inhibitory constants of the theaflavins against DXR.

	IC_50_ (μM)	NADPH		DXP	
		*K*i (μM)	Kinetics	*K*i (μM)	Kinetics
TF	162.1 ± 3.6	83.1 ± 4.3		107.3 ± 3.9	
TF3G	29.2 ± 2.6	24.7 ± 3.2		30.2 ± 2.6	
TF3′G	23.7 ± 2.9	22.4 ± 2.1	Uncompetitive	22.2 ± 3.1	Noncompetitive
TF3,3′G	14. 9 ± 2.1	18.2 ± 2.3		13.3 ± 2.4	
Fosmidomycin	0.27 ± 0.06				

**Table 3:** Influence of the presence of 0.01% Triton X-100 on the inhibition of *E. coli* DXR with the theaflavins and baicalein.

	Conc (μM)	Inhibition (%)		Conc (μM)	Inhibition (%)	
		No Triton	Triton		No Triton	Triton
TF	150	45.3 ± 3.4	43.9 ± 3.6	250	63.1 ± 2.4	62.2 ± 2.9
TF3G	30	52.6 ± 3.2	52.7 ± 2.4	50	69.6 ± 2.7	67.9 ± 3.4
TF3′G	20	41.3 ± 2.9	42.5 ± 3.3	40	70.3 ± 3.4	70.7 ± 3.3
TF3,3′G	15	47.1 ± 3.1	45.7 ± 2.8	30	78.7 ± 3.3	78.3 ± 3.2
Baicalein	50	37.6 ± 4.7	5.1 ± 3.9	100	63.5 ± 2.9	6.5 ± 4.6

**Table 4:** Dynamic Light Scattering of the theaflavins in 50 mM Tris-HCl buffer (pH 7.4).

	Conc (μM)*	Without DXR		With 4 μg/mL DXR	
		Count Rate (KHz)	Size (nm)	Count Rate (KHz)	Size (nm)
TF	500	2.2 ± 0.4	N/A**	5.8 ± 0.4	N/A
TF3G	100	2.6 ± 0.5	N/A	4.6 ± 0.5	N/A
TF3′G	100	1.4 ± 0.5	N/A	5.2 ± 0.4	N/A
TF3,3′G	70	2.2 ± 0.4	N/A	4.8 ± 0.4	N/A
Baicalein***	200	2.6 ± 0.5	N/A	296 ± 19.7	429.2 ± 84.7
Blank	—	2.6 ± 0.5	N/A	4.2 ± 0.4	N/A

^*^The assay mixture contained the theaflavins, 2 mM VC, and 2%DMSO (v/v); Blank comprised 2% DMSO (v/v) and 2 mM VC;

^**^N/A: not available.

^***^Baicalein was used as a positive control.

## References

[b1] Rohmer M. In Comprehensive Natural Products II: Chemistry and Biology (eds Mander L. & Liu H. W.) 517–556 (Oxford, 2010).

[b2] Singh N., Cheve G., Avery M. A. & McCurdy C. R. Targeting the MEP pathway for novel antimalarial, antibacterial and herbicidal drug discovery: inhibition of DXR enzyme. Curr. Pharm. Design 13, 1161–1177 (2007). 10.2174/138161207780618939 17430177

[b3] Jomaa H. . Inhibitors of the nonmevalonate pathway of isoprenoid biosynthesis as antimalarial drugs. Science 285, 1573–1576 (1999). 10477522 10.1126/science.285.5433.1573

[b4] Missinou M. A. . Fosmidomycin for malaria. Lancet 360, 1941–1942 (2002). 12493263 10.1016/S0140-6736(02)11860-5

[b5] Kaiser J. . Anti-malarial drug targets: Screening for inhibitors of IspC protein in Mediterranean plants. Phytomedicine 14, 242–249 (2007). 17293098 10.1016/j.phymed.2006.12.018

[b6] Friedman M. Overview of antibacterial, antitoxin, antiviral, and antifungal activities of tea flavonoids and teas. Mol. Nutr. Food Res. 51, 116–134 (2007). 17195249 10.1002/mnfr.200600173 PMC7168386

[b7] Von Staszewski M., Pilosof A. M. R. & Jagus R. J. Antioxidant and antimicrobial performance of different Argentinean green tea varieties as affected by whey proteins. Food Chem. 125, 186–192 (2011).

[b8] Quideau S., Deffieux D., Douat-Casassus C. & Pouységu L. Plant polyphenols: chemical properties, biological activities, and synthesis. Angew. Chem. Int. Ed. 50, 586–621 (2011). 10.1002/anie.201000044 21226137

[b9] de Oliveira A., Prince D., Lo C. Y., Lee L. H. & Chu T. C. Antiviral activity of theaflavin digallate against herpes simplex virus type 1. Antivir. Res. 118, 56–67 (2015). 25818500 10.1016/j.antiviral.2015.03.009 PMC7113870

[b10] Halder B., Pramanick S., Mukhopadhyay S. & Giri A. K. Anticlastogenic effects of black tea polyphenols theaflavins and thearubigins in human lymphocytes *in vitro*. Toxicol. In. vitro. 20, 608–613 (2006). 16314069 10.1016/j.tiv.2005.10.010

[b11] Stoner G. D. & Mukhtar H. Polyphenols as cancer chemopreventive agents. J. Cell. Biochem. 59, 169–180 (1995). 10.1002/jcb.240590822 8538195

[b12] Betts J. W., Kelly S. M. & Haswell S. J. Antibacterial effects of theaflavin and synergy with epicatechin againstclinical isolates of Acinetobacter baumannii and Stenotrophomonas maltophilia. Int. J. Antimicrob. Ag. 38, 421–425 (2011). 10.1016/j.ijantimicag.2011.07.006 21885260

[b13] Friedman M., Henika P. R., Levin C. E., Mandrell R. E. & Kozukue N. Antimicrobial activities of tea catechins and theaflavins and tea extracts against Bacillus cereus. J. Food Prot. 6, 354–361 (2006). 10.4315/0362-028x-69.2.354 16496576

[b14] Su Y. L., Leung L. K., Huang Y. & Chen Z. Y. Stability of tea theaflavins and catechins. Food Chem. 83, 189–195 (2003).

[b15] Haymond A. . Kinetic Characterization and Allosteric Inhibition of the Yersinia pestis MEP Synthase. Plos One 9, e106243 (2014). 25171339 10.1371/journal.pone.0106243 PMC4149570

[b16] Tritsch D., Zinglé C., Rohmer M. & Grosdemange-Billiard C. Flavonoids: True or promiscuous inhibitors of enzyme? The case of deoxyxylulose phosphate reductoisomerase. Bioorg. Chem. 59, 140–144 (2015). 25800132 10.1016/j.bioorg.2015.02.008

[b17] Feng B. Y. & Shoichet B. K. A detergent-based assay for the detection of promiscuous inhibitors. Nat. Protoc. 1, 550–553 (2006). 17191086 10.1038/nprot.2006.77 PMC1544377

[b18] McGovern S. L., Helfand B. T., Feng B. & Shoichet B. K. A specific mechanism of nonspecific inhibition. J. Med. Chem. 46, 4265–4272 (2003). 13678405 10.1021/jm030266r

[b19] Zinglé C., Tritsch D., Grosdemange-Billiard C. & Michel R. Catechol-rhodanine derivatives: Specific and promiscuous inhibitors of Escherichia coli DXR. Bioorg. Med. Chem. 22, 3713–3719 (2014). 24890653 10.1016/j.bmc.2014.05.004

[b20] Seidler J., McGovern S. L., Doman T. N. & Shoichet B. K. Identification and prediction of promiscuous aggregating inhibitors among known drugs. J. Med. Chem. 46, 4477–4486 (2003). 14521410 10.1021/jm030191r

[b21] Pohjala L. & Tammela P. Aggregating behavior of phenolic compounds -a source of false bioassay results? Molecules 17, 10774–10790 (2012). 22960870 10.3390/molecules170910774 PMC6268869

[b22] Cushnie T. P. T. & Lamb A. J. Recent advances in understanding the antibacterial properties of flavonoids. Int. J. Antimicrob. Ag. 38, 99–107 (2011). 10.1016/j.ijantimicag.2011.02.014 21514796

[b23] McGovern S. L. & Shoichet B. K. Kinase inhibitors: not just for kinases anymore. J. Med. Chem. 46, 1478–1483 (2003). 12672248 10.1021/jm020427b

[b24] Rishton G. M. Nonleadlikeness and leadlikeness in biochemical screening. Drug Discov. Today 8, 86–96 (2003). 12565011 10.1016/s1359644602025722

[b25] Shoichet B. K. Screening in a spirit haunted world. Drug Discov. Today 11, 607–615 (2006). 16793529 10.1016/j.drudis.2006.05.014 PMC1524586

[b26] Koppisch A. T., Fox D. T., Blagg B. S. J. & Poulter C. D. E. coli MEP synthase: steady-state kinetic analysis and substrate binding. Biochemistry 41, 236–243 (2002). 11772021 10.1021/bi0118207

[b27] Li H., Tian J., Wang H., Yang S. Q. & Gao W. Y. An Improved Preparation of D-Glyceraldehyde 3-Phosphate and Its Use in the Synthesis of DXP. Helv. Chim. Acta. 93, 1745–1750 (2010).

[b28] Li H., Tian J., Sun W., Qin W. & Gao W. Y. Mechanistic insights into DXR, a key enzyme of the MEP terpenoid biosynthetic pathway. FEBS. J. 280, 5896–5905 (2013). 24010408 10.1111/febs.12516

[b29] Hu Y., Wang X. J., Li H. & GAO W. Y. Determination of steady-state kinetic parameters of DXS by pre-column derivatization HPLC using 2, 4-Dinitrophenylhydrazine as derivative reagent. Chin. J. Anal. Chem. 40, 1859–1864 (English version) (2012).

[b30] Sweeney A. M. . The crystal structure of E. coli DXR in a ternary complex with the antimalarial compound fosmidomycin and NADPH reveals a tight-binding closed enzyme conformation. J. Mol. Biol. 345, 115–127 (2005). 15567415 10.1016/j.jmb.2004.10.030

