# Supplementary material for: Antimicrobial mechanism of theaflavins: They target 1-deoxy-D-xylulose 5-phosphate reductoisomerase, the key enzyme of the MEP terpenoid biosynthetic pathway
Source: Sci Rep. 2016 Dec 12;6:38945. doi: 10.1038/srep38945 (PMC5150241; doi:10.1038/srep38945)
Supplement: Supplementary Information [file srep38945-s1.pdf]

Supplementary Material

Antimicrobial mechanism of theaflavins: They target 1-deoxy-D-xylulose 5-phosphate reductoisomerase, the key enzyme of the MEP terpenoid biosynthetic pathway

Xian Hui, Qiao Yue, Dan-Dan Zhang, Heng Li, Shao-Qing Yang and Wen-Yun Gao*

1. Stability of the theaflavins under the DXR assay conditions

In order to evaluate the stability of the theaflavins under the DXR assay conditions, each compound was diluted into 50 mM Tris-HCl buffer (pH 7.4) and incubated at 37 ^{o}C for 35 min in the absence and presence of 2 mM VC before they were determined by HPLC. The HPLC profiles were listed in Fig. S1. From the figure we are able to see that in the presence of 2 mM VC, the four theaflavin compounds are stable because the peak area of each compound remains almost unchanged after incubation (B). But they are unstable in the absence of VC because the peak areas of all four compounds reduced by around 40% after incubation (C).

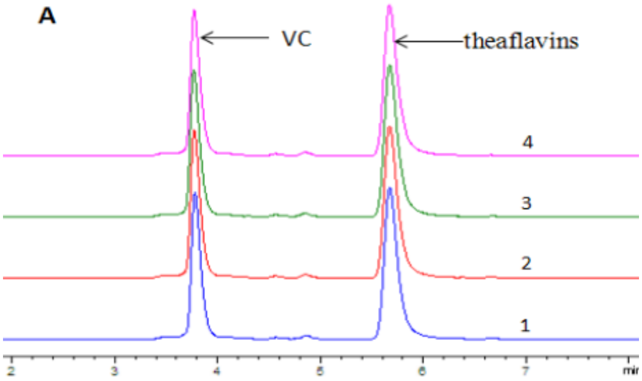

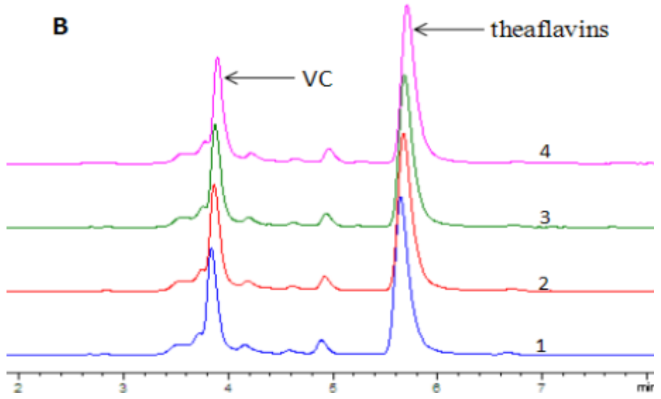

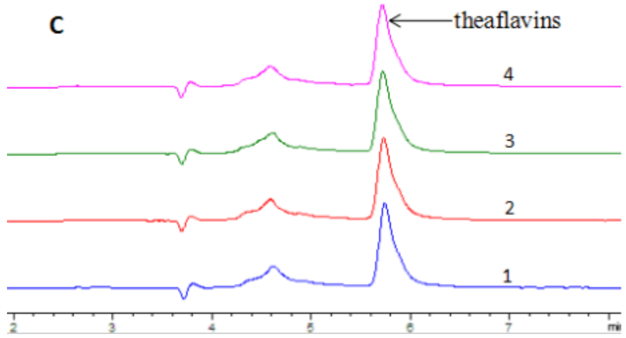

Figure S1. The stabilitiy of the theaflavin compounds under DXR assay conditions. **A.** Mixtures of VC and individual theaflavins in 50 mM Tris-HCl buffer (pH 7.4) before incubation; **B.** Mixtures of VC and individual theaflavins in same buffer after incubated at 37 ^{o}C for 35 min; **C.** Individual theaflavins in same buffer after incubated at 37 ^{o}C for 35 min. In **A** and **B**. Line 1, TF (100 μM) plus VC (2 mM); Line 2, TF3G (100μM) plus VC (2 mM); Line 3, TF3'G (100μM) plus VC (2 mM); Line 4, TF3,3'G (100μM) plus VC (2 mM). In **C**. Line 1, TF (100 μM); Line 2, TF3G (100μM); Line 3, TF3'G (100μM); Line 4, TF3,3'G (100μM). HPLC conditions: Column, Shim-pack VP-ODS column (250 × 4.6 mm, 4.6μm). Detection wavelength: 280 nm. The mobile phase consisted of 60% solvent A [2% acetic acid in water (v/v)] and 40% solvent B (acetonitrile). Flow rate: 0.7 mL/min. Column temperature: 25 ^{o}C.

2. Evaluation of the pre-column derivatization HPLC method for the quantification of DXP.

2.1. Derivatization of 1-deoxy-D-xylulose-5-phosphate with 2,4-dinitrophenylhydrazine.

Carbonyl containing compounds can be determined by HPLC through pre-column derivatization with 2,4-Dinitrophenylhydrazine (DNPH). Carbonyl can react with phenylhydrazine under acidic condition via nucleophilic addition to generate phenylhydrazone which possesses characteristic UV absorption at about 360 nm. In the conversion of 1-deoxy-D-xylulose 5-phosphate (DXP, **1**, Fig. S2) to 2-methyl-D-erythritol 4-phosphate (MEP, **2**) catalyzed by 1-deoxy-D-xylulose 5-phosphate reductoisomerase (DXR), compound **1** is a ketose phosphate that may be derivatized with DNPH. In order to perform the reaction under a relatively mild condition and facilitate HPLC analysis, the residual **1** was first dephosphorylated with calf intestine alkaline phosphatase (CIAP) to the free sugar compound DX (**3**). Subsequently, **3** reacted with DNPH in acidic medium to form 1-deoxy-*D*-xylulose-dinitrophenylhydrazone (DX-DNPH, **4**) which can be quantified by HPLC[1]. The reaction principle is shown in Fig. S2.

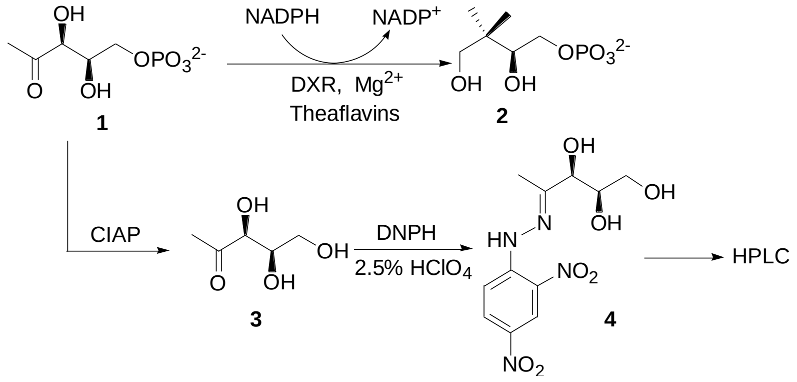

Figure S2. Derivatization of 1-deoxy-D-xylulose-5-phosphate with 2,4-dinitrophenylhydrazine. After the DXR assay, the residual DXP was dephosphorylated by AP in Tris-HCl buffer (pH 9.5) at 37°C for 120 min, derivatized with DNPH (in about 1% perchloric acid, V/V) at 37 °C for another 45 min, and then centrifuged and used for HPLC analysis.

2.2. HPLC analysis.

In order to determine whether the addition of theaflavins and VC to DXR assay mixture could interfere with the quantification of DXP, we chromatographed assay mixtures (50 mM Tris-HCl

buffer (pH 7.4), 5 mM $MgCl_2$, 2% (W/V) DMSO) containing VC and each of the four theaflavin compounds after the mixtures had been treated with AP and DNPH. The HPLC profiles were listed in Fig. S3. The results show that after AP and DNPH treatment, only DXP gives a peak at a retention time about 8 min (DX-DNPH), no peak of the theaflavins and VC are detectable at all. The reason could be that the theaflavins and VC decomposed completely under the relatively harsh conditions for dephosphorylation (pH 9.5, 120 min) and DNPH derivatization (about 1% perchloric acid, 45 min). Fortunately, the decomposed theaflavins and VC do not interfere with the determination of DXP. Thus, we are able to conclude that the pre-column derivatization HPLC method is suitable for the quantification of DXP.

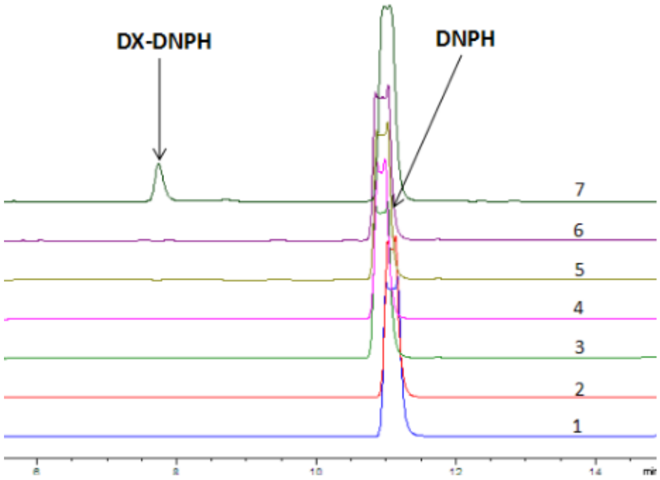

Figure S3. Evaluation of the pre-column derivatization HPLC method for the quantification of DXP. HPLC conditions: Column, Shim-pack VP-ODS column (250 × 4.6 mm, 4.6μm). Mobile phase: acetonitrile-water gradient: 0 min, 40% acetonitrile; 17 min, 80% acetonitrile; 18-20 min, 40% acetonitrile. Detection wavelength: 360 nm. Flow-rate: 0.7 mL/min. Column temperature: 30 ^{o}C. Line 1, DNPH; Line 2, VC; Line 3, TF (250μM) and VC (2 mM); Line 4, TF3G (100μM) and VC (2 mM); Line 5, TF3'G (100μM) and VC (2 mM); Line 6, TF3,3'G (100μM) and VC (2 mM); Line 7, DNPH derivatized DXP (1mM).

3. The inhibition kinetics of the theaflavins against DXR

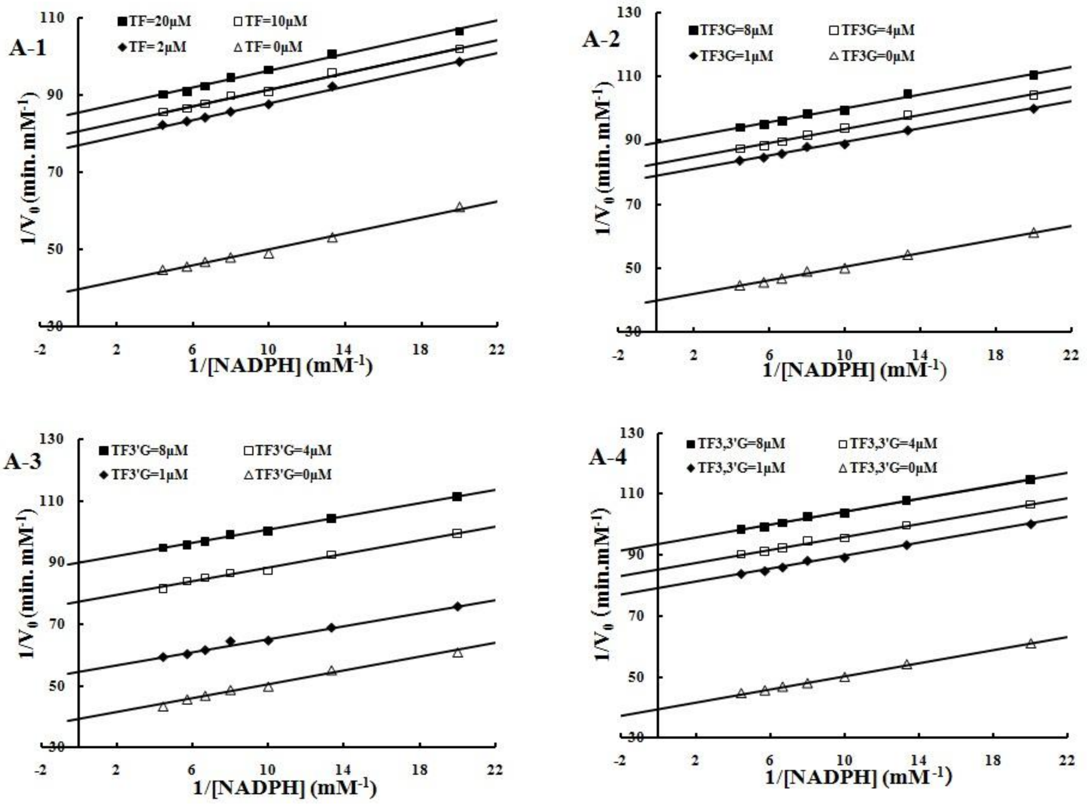

Figure S4. Lineweaver–Burk plots of DXR with respect to NADPH in the absence and presence of TF (**A-1**), TF3G (**A-2**), TF3’G (A-**3**), and TF3,3’G (**A-4**). Assay mixtures comprised 50 mM tris-HCl (pH 7.4), 5 mM $MgCl_2$, 2 mM VC, the theaflavins (TF: 0, 2, 10, 20 μM; the other three compounds: 0, 1, 4, or 8 μM), 1 mM DXP and 2 μg/mL DXR in a final volume of 100 μL. Then, NADPH (final conc. 0.05 to 0.275 mM) was added, and incubation was performed for 10 min at 37°C before the reaction mixtures were hydrolyzed with AP, derivatized with DNPH, and analyzed using the HPLC method. (**A-1**) DXR inhibition kinetics of TF; (**A-2**) DXR inhibition kinetics of TF3G; **(A-3**) DXR inhibition kinetics of TF3’G; **(A-4**) DXR inhibition kinetics of TF3,3’G.

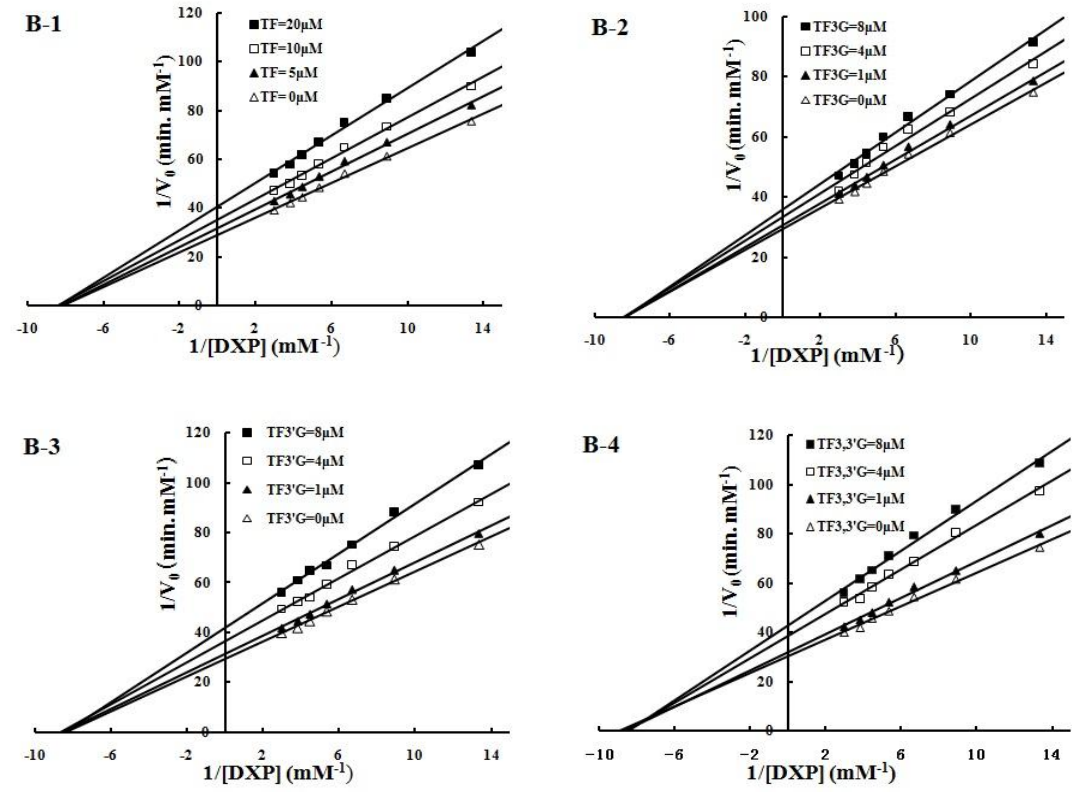

Figure S5. Lineweaver–Burk plots of DXR with respect to DXP in the absence and presence of TF (**B-1**), TF3G (**B-2**), TF3’G (**B-3**), and TF3,3’G (**B-4**). Assay mixtures comprised 50 mM tris-HCl (pH 7.4), 5 mM $MgCl_2$, 2 mM VC, the theaflavins (TF: 0, 5, 10, 20 μM; the other three compounds: 0, 1, 4, or 8 μM), 0.5 mM NADPH, and 2 μg/mL. DXR in a final volume of 100 μL. Then, DXP (final conc. 0.075 to 0.3375 mM) was added, and incubation proceeded for 10 min at 37°C before the reaction mixtures were hydrolyzed with AP, derivatized with DNPH, and analyzed using the HPLC method. (**B-1**) DXR inhibition kinetics of TF; (**B-2**) DXR inhibition kinetics of TF3G; (**B-3**) DXR inhibition kinetics of TF3’G; (**B-4**) DXR inhibition kinetics of TF3,3’G.

4. Docking results.

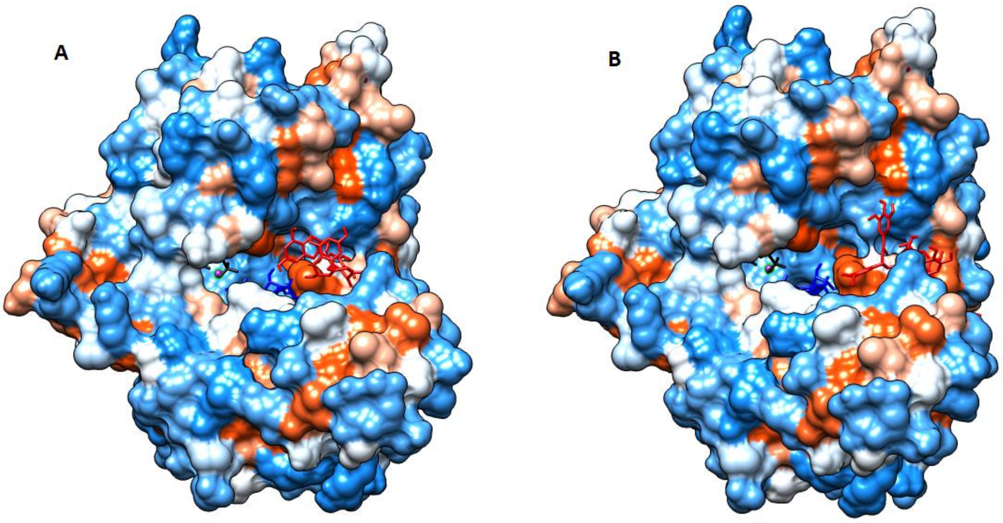

Figure S6. A. The binding mode of TF3,3'G and the substrates on DXR; **B**. The binding mode of TF and the substrates on DXR. The enzyme (monomer) is shown as the surface, while the structures of the substrates and the inhibitors are shown as sticks. The inhibitors are shown as red sticks, and DXP and NADPH are the black and blue sticks, respectively; Mg^{2+} is shown as purple balls.

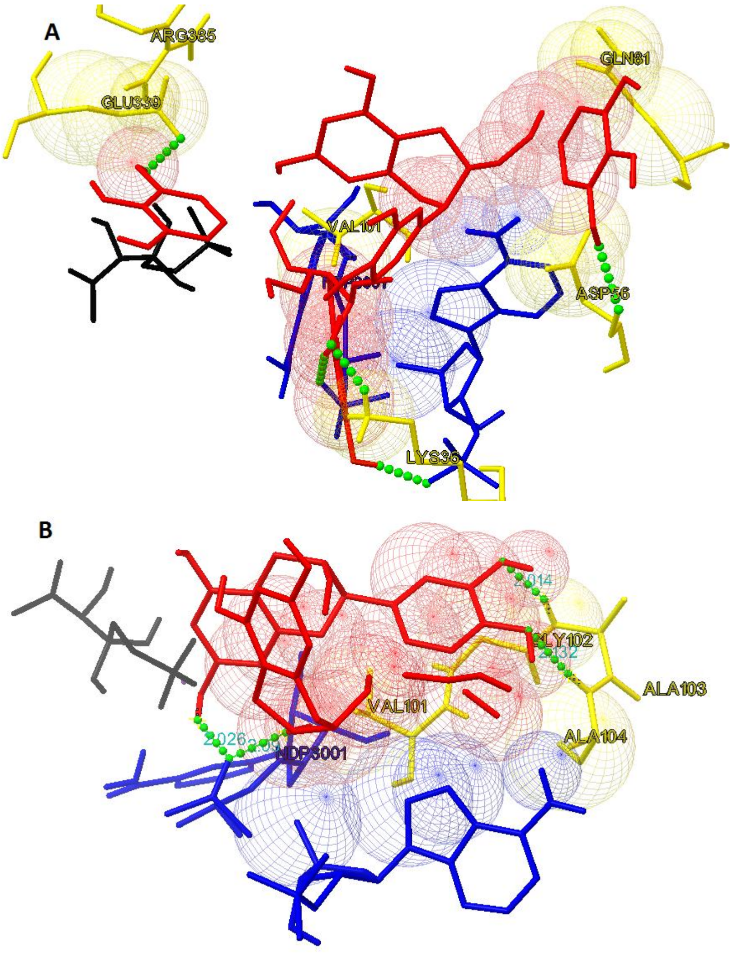

Figure S7. **A**. The close-up views of interactions between the inhibitors, substrates, and residues. A. The inhibitor is TF3,3'G; **B**. The inhibitor is TF. The inhibitors are shown in red; the residues, DXP, NADPH, and Mg^{2+} are shown in yellow, black, blue, and purple, respectively. The wire balls represent close contact, yellow: between the inhibitor and the residues; blue: between the inhibitor and NADPH; red: between the inhibitor and NADPH/residues/DXP. The green bead wires represent the hydrogen bonds.

References

1. Hu, Y., Wang, X. J., Li, H., Gao, W. Y., 2012. Determination of steady-state kinetic parameters of DXS by pre-column derivatization HPLC using 2, 4-Dinitrophenylhydrazine as derivative reagent. *Chinese Journal of Analytical Chemistry, 40*, 1859-1864. (English version)
